# A preclinical model for phenogroup 3 HFpEF

**DOI:** 10.18632/aging.102102

**Published:** 2019-07-15

**Authors:** Keyvan Yousefi, Julian C. Dunkley, Lina A. Shehadeh

**Affiliations:** 1 Department of Molecular and Cellular Pharmacology, University of Miami Leonard M. Miller School of Medicine, Miami, FL 33136, USA; 2 Interdisciplinary Stem Cell Institute, University of Miami Leonard M. Miller School of Medicine, Miami, FL 33136, USA; 3 Department of Medicine, Division of Cardiology, University of Miami Leonard M. Miller School of Medicine, Miami, FL 33136, USA; 4 Vascular Biology Institute, University of Miami Leonard M. Miller School of Medicine, Miami, FL 33136, USA; 5 Peggy and Harold Katz Family Drug Discovery Center, University of Miami Leonard M. Miller School of Medicine, Miami, FL 33136, USA

**Keywords:** Alport syndrome, HFpEF, mouse model, osteopontin, mitochondria

Heart failure with preserved ejection fraction (HFpEF) is a heterogeneous syndrome seen in a predominantly elderly population, with multiple comorbidities including but not limited to: hypertension (systemic and pulmonary), chronic kidney disease (CKD), obesity, diabetes mellitus (DM) and coronary artery disease. Due to this intricate pathophysiology, definitive preclinical models are lacking and conventional HF therapeutic regimens fail in definitively treating HFpEF. This conundrum creates the need to classify individual patients into groups with certain cardiac and extracardiac abnormalities to successfully address HFpEF phenotypic diversity. To this end, Shah et al. [1]. proposed three HFpEF phenogroups based on clinical variables, physical characteristics, laboratory data, ECG parameters, and echocardiographic parameters. Phenogroup 1 comprises of patients with moderate diastolic dysfunction. Phenogroup 2 represents obese, diabetic patients with a high prevalence of obstructive sleep apnea and the highest degree of left ventricular relaxation impairment. Phenogroup 3 includes older patients with CKD, myocardial remodeling, arrhythmia, pulmonary hypertension (PH), and right ventricular (RV) dysfunction [1].

**Figure 1:**
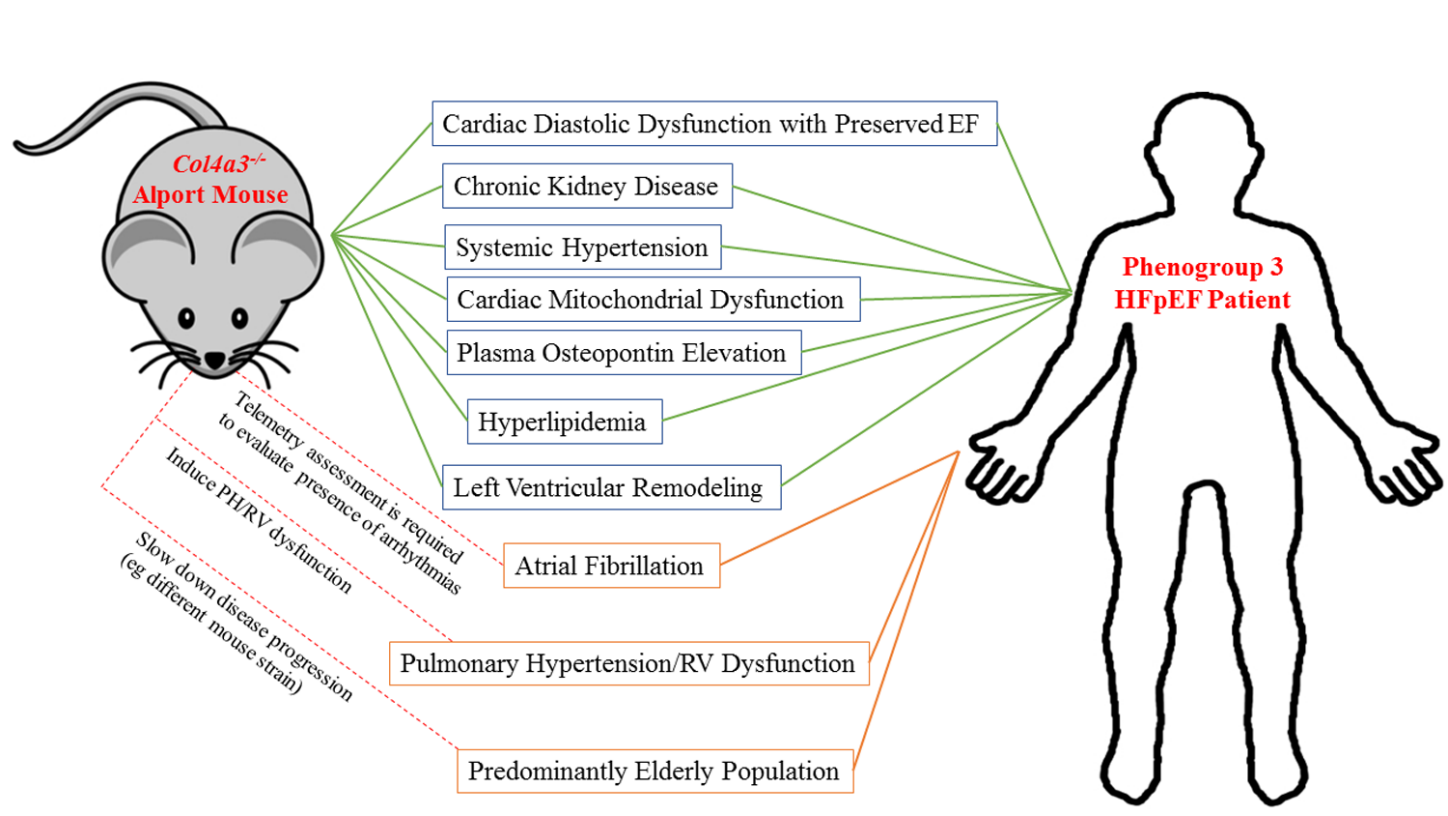
**Engineering the Alport mouse to become a closer model for phenogroup 3 HFpEF patients.** The Alport mouse represents multiple cardiac and extracardiac features of phenogroup 3 HFpEF patients (bound by green lines). Other HFpEF comorbidities not displayed or investigated in the Alport mouse (bound by orange lines) need to be addressed in order to engineer the mouse to better mimic phenogroup 3 HFpEF patients.

Our group recently introduced the *Col4a3^-/-^* mouse model of Alport nephropathy as a viable preclinical HFpEF model by recapitulating a range of HFpEF-related cardiac (diastolic dysfunction, mitochondrial dysfunction and remodeling) and extracardiac abnormalities (CKD, hypertension, dyslipidemia), summarized in Figure 1 [2]. An attractive fact about this model is that the Alport mice develop all of these HFpEF features spontaneously without the need for any pharmacological or surgical interventions at very early ages (at 2months of age for Alport mice on 129J or mixed B6/129J/Balbc (triple strain) background). However, Alport mice have a short lifespan and the development of HFpEF at young ages may be considered a disadvantage, as HFpEF is an elderly dominated disease. In addition, the very short lifespan of the mouse (despite being a useful feature for high throughput preclinical studies), is considered a disadvantage considering that HFpEF is generally a slowly progressive disease. Nonetheless, even with its limitations, the Alport mouse is a starting point towards a more representative preclinical model of phenogroup 3 HFpEF patients [1]. It is important to note that while obesity and DM are most common in phenogroup 2, phenogroup 3 included 37% and 34% of patients with these conditions, respectively. Therefore the Alport mouse may be considered to represent phenogroup 3 in the absence of obesity and DM. The genetic background significantly impacts the progression rate of CKD and life span in Alport mice [3]. For example, while Alport mice on pure 129J background develop end-stage renal disease (ESRD) at about 2 months of age, it takes more than 6 months for the C57/Bl6 strain to progress to ESRD and eventually die. While this does not perfectly replicate the effect of aging on HFpEF, it is certainly a vast improvement, with more opportunity for therapeutic intervention and data collection. In our previous study, we showed that similar to the 129J strain, Alport mice on mixed background develop ESRD close to 2 months of age [4]. We also showed that Alport mice on 129J or mixed strain develop diastolic dysfunction; however, contrary to the mixed-strain animals, 129J mice do not present cardiac hypertrophy [2]. Therefore, strain manipulation is an attractive area for further investigation on the use of the Alport mouse model for HFpEF. These differences could be viewed as an opportunity to trim and perfect some of its potential limitations as a representative model [2]. For instance, the use of B6 strain may allow for a slower progression of HFpEF while preserving other HFpEF features.

By taking advantage of the existing preclinical models, it is plausible to introduce other missing cardiac or extracardiac features in the Alport HFpEF model. Specifically, while we showed a lack of PH in Alport mice [2], the occurrence of arrhythmias in this model is not yet investigated. Phenogroup 3 was shown, by a large margin, to have the highest prevalence of atrial fibrillation (AF) among HFpEF patients [1]. If telemetry demonstrates a propensity of the Alport mice to develop AF, this would provide further evidence of its likeness to phenogroup 3. Otherwise, to engineer the Alport mouse to become closer to a phenogroup 3 HFpEF phenotype, further pharmacological interventions could be employed to induce PH/RV dysfunction (Figure 1). A possible option to induce PH and RV dysfunction is administration of Sugen (SU5416), a vascular endothelial growth factor receptor-2 (VEGFR) blocker that can trigger lung endotheliopathy and ultimately lead to PH and RV dysfunction [5,6]. Thus, the potential exists for the Alport mouse to provide relevant data pertaining to phenogroup 3, which features the most severe manifestation of the disease as well as the highest mortality risk [1] (based on the Meta-Analysis Global Group in Chronic Heart Failure Risk Score) among the three groups.

While it has not been fully characterized, current evidence supports a long-standing theory that mitochondrial dysfunction is an important contributor towards both the normal aging process as well as age-related diseases [7]. There is also strong evidence suggesting that mitochondrial dysfunction is an integral part of HFpEF pathophysiology [8] which also affects a predominantly elderly population; for this reason there are numerous trials currently underway which aim to explore mitochondrial function as a therapeutic target for this disease. We demonstrated that osteopontin induces mitochondrial dysfunction, promoting a HFpEF phenotype in the Alport mouse [2]. Therefore, the Alport mouse, especially on slow progressing strains, (e.g. C57BL/6) and if the mitochondrial and diastolic dysfunction phenotypes persist, may mimic the aging mitochondrial profile of HFpEF patients (Figure 1).
